# Core Neuropsychological Measures for Obesity and Diabetes Trials: Initial Report

**DOI:** 10.3389/fpsyg.2020.554127

**Published:** 2020-09-24

**Authors:** Kimberlee D’Ardenne, Cary R. Savage, Dana Small, Uku Vainik, Luke E. Stoeckel

**Affiliations:** ^1^Department of Psychology, Arizona State University, Tempe, AZ, United States; ^2^Department of Psychology, University of Nebraska–Lincoln, Lincoln, NE, United States; ^3^Center for Brain, Biology and Behavior, Department of Psychology, University of Nebraska–Lincoln, Lincoln, NE, United States; ^4^Modern Diet and Physiology Research Center (MDPRC), Department of Psychiatry, Yale School of Medicine, Yale University, New Haven, CT, United States; ^5^Department of Psychology, Yale University, New Haven, CT, United States; ^6^Institute for Diabetes Research and Metabolic Diseases of the Helmholtz Center Munich at the University of Tübingen, Tübingen, Germany; ^7^Institute of Psychology, Faculty of Social Sciences, University of Tartu, Tartu, Estonia; ^8^Department of Neurology and Neurosurgery, Faculty of Medicine, McGill University, Montreal, QC, Canada; ^9^National Institute of Diabetes and Digestive and Kidney Diseases, National Institutes of Health, Bethesda, MD, United States

**Keywords:** executive function, cognitive control, reward, motivation, decision-making, memory, sensation, perception

## Abstract

Obesity and diabetes are known to be related to cognitive abilities. The Core Neuropsychological Measures for Obesity and Diabetes Trials Project aimed to identify the key cognitive and perceptual domains in which performance can influence treatment outcomes, including predicting, mediating, and moderating treatment outcome and to generate neuropsychological batteries comprised of well-validated, easy-to-administer tests that best measure these key domains. The ultimate goal is to facilitate inclusion of neuropsychological measures in clinical studies and trials so that we can gather more information on potential mediators of obesity and diabetes treatment outcomes. We will present the rationale for the project and three options for the neuropsychological batteries to satisfy varying time and other administration constraints. Future directions are discussed. Preprint version of the document is available at https://osf.io/preprints/nutrixiv/7jygx/.

## Introduction

Obesity and diabetes are associated with an array of cognitive and perceptual differences, some of which can be classified as mild impairments. Many of these differences are likely pre-existing and contribute to initial risk (e.g., Davis, 2009; Silveira et al., 2012), while others worsen or emerge as a consequence of the pathophysiological processes associated with obesity and diabetes. Determining the mechanisms and disentangling cause from consequence is a focus of ongoing research into the cognitive differences associated with obesity and diabetes. Typical treatment outcomes for obesity include change in body mass index (BMI), weight, and fat percentage; and change in HbA1C for (type 2) diabetes. For instance, lower cognitive ability in childhood is associated with weight gain in adulthood via diet and education levels (Chandola et al., 2006). Cognitive dysfunction, including executive dysfunction (e.g., impulsivity), poor memory, and decreased interoceptive awareness - among others - could hamper weight loss efforts (e.g., Koritzky et al., 2015; Schäfer et al., 2019).

This project aimed to identify the key cognitive and perceptual domains in which performance can influence treatment outcomes. Possible influences include predicting, mediating, and moderating treatment outcomes. The project serves as a basis for generating neuropsychological batteries composed of well-validated, easy-to-administer tests that best measure these key domains. The work builds upon several systematic reviews and meta analyses that link many different cognitive domains cross-sectionally to obesity (see Vainik et al., 2013; Wu et al., 2016; Emery and Levine, 2017; Yang et al., 2018). Typical effect sizes vary from *r* = 0.10 to 0.20 (Emery and Levine, 2017; Yang et al., 2018). However, not all cognitive tests from those analyses should be included, as cognitive tests also correlate with each other (Sharma et al., 2014; Eisenberg et al., 2019). Also, the meta-analyses may not include all tests that should be linked with obesity and diabetes. We therefore sought to have experts synthesize the previous findings outlined in the above-mentioned summaries, as well as highlight less-studied domains that should be included in future studies on obesity and diabetes.

Notably, there are more research domains than neuropsychology related to obesity and diabetes. One resource for additional measures and guidance is the ADOPT initiative (MacLean et al., 2018). We have also listed several approaches in Appendix B, such as personality tests and polygenic scores. Other data commonly used for clinical studies that include neuropsychological measures such as information on age, sex, education, and socioeconomic status, often used to develop normative data for performance evaluation, should be considered.

Here, the Core Neuropsychological Measures for Obesity and Diabetes Trials Project (CoreNP) expert group developed three options for the neuropsychological batteries to satisfy varying time and other administration constraints. *The ultimate goal is to facilitate inclusion of cognitive measures in clinical studies and trials so that we can gather more information on potential mediators of obesity and diabetes treatment outcomes*. To achieve this goal, the recommended batteries could be used in ongoing and future obesity and diabetes trials so that the relationship between cognition (broadly) and obesity and diabetes can be better understood, in turn identifying the most promising cognitive domains to include as predictors, mediators, and moderators in trials and as targets for intervention. Standardizing neuropsychological batteries would also facilitate data sharing and analyzing aggregate data across multiple studies.

For example, diet and physical activity are known to impact cognitive function and are both strongly associated with diabetes and obesity (Davidson et al., 2013; Freeman et al., 2014; Morris et al., 2015; Espeland et al., 2018). While we did not consider interactions between diet/physical activity and cognition in test selection, such interactions likely play an important modulatory role in treatment outcome. However, while most obesity and diabetes trials include measures of diet and physical activity, few also measure cognition. The addition of cognitive measures should therefore facilitate new insights into whether cognitive changes contribute to the efficacy of interventions that focus on diet and physical activity. For instance, a recent longitudinal study suggests that decline in cognitive ability precedes later decline in physical activity (Cheval et al., 2020).

## Workshop Charge

The specific charge of the CoreNP expert group was to recommend one or two stand-alone neuropsychological tasks that spanned major cognitive domains (Table 1) and met many or most of the highest-priority criteria, which are listed in Table 2 and Appendix A. The psychometric properties of a task, especially task test–retest reliability, were of particular importance (further detail on test–retest reliability in Appendices B,E). The Executive Committee (Appendix C) prioritized the criteria, which were intended to serve as guidance for groups with expertise in six major cognitive domains: (1) cognitive control/executive function, (2) reward/motivation, (3) attention/working memory, (4) learning/memory, (5) sensation/perception, and (6) decision-making. Because the state of knowledge varies considerably across domains, the relative weight or importance of each criterion or set of criteria varied across domain groups. The groups were asked to indicate (a) the criteria considered in making task recommendations and the relative weight of each criterion that led to the final recommendations, and (b) the evidence base used to justify the recommendations.

**TABLE 1:** Summary of neuropsychological domains.

**Domain**	**Definition**
Executive function/cognitive control	Cognitive control/executive function is a multifaceted construct including many sub-domains (e.g., cognitive flexibility and response inhibition) related to the ability to coordinate thought and action and direct it toward obtaining goals (Miller and Wallis, 2009).

Reward/motivation	A reward is an environmental stimulus that is able to elicit approach responses. A central feature of reward is reinforcement or the tendency of certain stimuli to strengthen learned stimulus-response tendencies (White, 1989). Motivation refers to the energization of behavior by, or the direction of behavior toward, positive stimuli (e.g., objects, events, and possibilities) (Elliot, 2006).

Attention/working memory	Attention refers to the ability to select stimuli and actions that are coherent with the goals of an organism (Corbetta et al., 2002). Working Memory is the active maintenance and flexible updating of goal/task relevant information (items, goals, strategies, etc.) in a form that has limited capacity and resists interference (https://www.nimh.nih.gov/research/research-funded-by-nimh/rdoc/working-memory-workshop-proceedings.shtml).

Learning/memory	Learning is the acquisition of skill or knowledge, while memory is the storage and retrieval of the skill or knowledge one has acquired (Kazdin, 2000).

Sensation/perception	Sensation is the process of detecting our internal and external environments. Perception occurs when sensory signals are interpreted and integrated in the central nervous system to produce a conscious experience (Goldstein, 2014).

Decision-making	The process of identifying and choosing alternatives based on the values, preferences and beliefs of the decision-maker (Kahneman and Tversky, 2000).

**TABLE 2:** High priority criteria used for task selection.

1	How strong is the evidence that the task has good overall psychometric characteristics for measuring individual differences (e.g., internal reliability, test–retest reliability, minimal floor and ceiling effects in healthy populations and clinically impaired populations, minimal practice effects, availability of alternate forms, longitudinal stability)?

2	How likely is it that the task will provide utility in predicting obesity and diabetes outcomes?

3	Is the measure useful as a predictor, moderator, mediator, or outcome? (address for all potential uses)

4	Does the measure show a cross-sectional association with Body Mass Index (BMI), obesity, insulin resistance, type 2 diabetes, physical activity, sedentary behavior, diet, caloric intake, adherence to diet and physical activity recommendations, and prescribed treatment?

5	Does the measure show group differences in BMI, obesity, insulin resistance, type 2 diabetes, physical activity, sedentary behavior, diet, caloric intake, adherence to diet and physical activity recommendations, and prescribed treatment?

6	Does the measure show a prospective association with BMI, obesity, insulin resistance, type 2 diabetes, physical activity, sedentary behavior, diet, caloric intake, adherence to diet and physical activity recommendations, and prescribed treatment?

7	Is the measure predictive of change in BMI, obesity, insulin resistance, type 2 diabetes, physical activity, sedentary behavior, diet, caloric intake, adherence to diet and physical activity recommendations, and prescribed treatment?

**TABLE 3:** Standard battery.

**Test**	**Version/source**	**Administration time**	**Scoring**
**General cognitive index**			
Visual pattern reasoning	https://www.testmybrain.org/tests/matrix_discontinuerule/index_numbered.html	7 min	Accuracy
Digit symbol matching	https://www.testmybrain.org/tests/DigSymbCoding/DSC.html	2 min	Number of correct matched pairs
**Executive function/cognitive control**			
Go/no-go	NIH Toolbox: https://scienceofbehaviorchange.org/measures/go-no-go-task/	4 min	*d*′; commission error rate (i.e., response on no-go trials)
Dimensional change card sort	NIH Toolbox: http://www.healthmeasures.net/explore-measurement-systems/nih-toolbox/intro-to-nih-toolbox/cognition	4 min	Accuracy score
**Learning/memory**			
Picture-sequence memory	NIH Toolbox: http://www.healthmeasures.net/explore-measurement-systems/nih-toolbox/intro-to-nih-toolbox/cognition	7 min	Accuracy
**Decision-making**			
Kirby delay discounting task, 27 Item	https://scienceofbehaviorchange.org/measures/kirby-delay-discounting-task/	5 min	Discount factor, *k*

The CoreNP expert group recommends three options to incorporate neuropsychological assessments in ongoing and future adult obesity and diabetes trials: (1) a 10-min General Cognitive Index (GCI); (2) a Standard Battery that includes the GCI; or (3) an Extended Neuropsychological Test Battery. The GCI is recommended as a “must” include, while the Standard and Extended batteries are recommended as “may” include because they require additional empirical support for their utility in predicting, mediating, or moderating outcomes in adult obesity and diabetes trials.

We note that the Standard Battery does not include tasks recommended for the reward/motivation or attention/working memory domains. Tasks from these domains were not included as there were insufficient data to support the psychometric properties of the task (reward/motivation) and/or the task administration time would extend the Standard Battery beyond 30 min (both reward/motivation and attention/working memory). Tests for these domains are included in the *Extended Battery* and are highly recommended. Some measures like those from the NIH Toolbox^1^, TestMyBrain.org, NIH Examiner^2^, and WAIS-IV^3^ may include a fee for some uses, but fees may also allow access to additional measures that may be of interest for obesity and diabetes trials. However, where possible, we selected measures without fees.

The *Standard Battery* includes the GCI and tests that meet the highest priority criteria. The Standard Battery can be administered in approximately 30 min. The Extended Battery includes the Standard Battery measures as well as high priority measures identified using the high priority criteria for task selection that were deemed promising but were not included in the Standard Battery either because the data supporting their relationship to treatment outcomes were insufficient or because the tests required too much time for preparation and administration. The *Extended Battery* will provide a more comprehensive assessment but will require approximately 80 min for administration.

Within this report, we list the currently available measures that are recommended for the GCI, Standard, and Extended Batteries. We also list tasks that were not included because they require further optimization, testing, and validation (Appendix D). To facilitate direct comparisons between tasks during selection, the expert group members rated each proposed task or measurement in the Task Recommendation Matrix using a scale of 1–5 (1 = no evidence, 3 = some evidence, 5 = strong evidence). Table 2 lists the criteria used for task selection. Additional criteria are included in Appendix A.

## General Cognitive Index

### Purpose

The goal of the General Cognitive Index (GCI) is to provide a brief (∼10 min) measure of global cognitive function for use in adult obesity and diabetes trials. The GCI provides a neuropsychological assessment that is sensitive to individual differences in overall cognition when a more comprehensive assessment is not feasible. The GCI also provides standard measures that can be used to interpret performance on other measures in the Standard and Extended Batteries, relative to overall cognitive ability. The GCI uses tests that are not highly dependent on English language skills, making it more appropriate for use across languages and cultures than language-dependent alternatives.

### Recommended Tests

The GCI uses two measures that capture individual differences in non-verbal reasoning and processing speed:

(1) Visual pattern reasoning (matrix reasoning format), 7 min.
(2) Digit symbol matching speed (digit symbol substitution format), 2 min.

Baseline performance on the matrix reasoning task can be used as a stable marker of cognitive ability that may predict outcome; while changes in performance on digit symbol matching may indicate that improved (or impaired) cognition may be contributing to the efficacy of an intervention. Along with GCI, we recommend measuring educational attainment, for instance by counting years of education or by using the International Standard Classification of Education Standard. These two measures are interchangeable (e.g., Rietveld et al., 2014, Supplementary Table S1). Education is closely related to general cognitive ability (Lee et al., 2018), and education may explain the effect of cognition on weight change (Chandola et al., 2006). Recommendations for additional data to collect can be found in the “Next Steps” section.

### Rationale for Test Selection

The matrix reasoning task assesses non-verbal or visual reasoning, general cognitive ability, and/or fluid intelligence (Lezak et al., 2012). When compared to other standard neuropsychological measures, matrix reasoning tests tend to load most highly on the so-called ‘g’ factor of general cognitive ability (Carroll, 1993). Measures of matrix reasoning are a reliable method of assessing general cognitive functioning and can be easily administered on digital devices such as computers, tablets, and smartphones. Poorer scores on matrix reasoning tests have been linked with poorer outcomes in obesity and diabetes (Dore et al., 2008; Feinkohl et al., 2014). As matrix reasoning scores tend to be relatively stable over time and less sensitive to state-related changes, matrix reasoning is often considered a premorbid IQ or “hold” test (Schoenberg et al., 2006; Tranel et al., 2008) and performance is less affected by changes in health or brain damage than other cognitive tests (Ryan et al., 2005). Matrix reasoning scores also provide a useful potential covariate for understanding specific cognitive mechanisms, where the researcher wants to control for variations due to general cognitive ability. The stability of matrix reasoning performance over time makes this measure a useful predictor (or moderator) of outcomes in studies. This test is not recommended as an outcome measure in obesity and diabetes intervention studies because performance is relatively insensitive to changes in health status and any given form of matrix reasoning can only be given once. Thus, changes in scores over time on the same test are difficult to interpret and not necessarily meaningful.

The digit symbol matching test can be administered using digital devices. This 2-min test uses a standard format digit symbol substitution procedure, but with a keyboard or touchscreen rather than written responses. The test measures processing speed and a range of cognitive functions including visual scanning, sustained attention, and short-term memory (Lezak et al., 2012). Scores on this measure are sensitive and reliable despite very brief administration times. Performance on this test is less related to education and general cognitive ability as measured by more stable trait-like measures (e.g., matrix reasoning). Instead, the digit symbol matching test is a very sensitive measure of many state variables, including changes in health and all or nearly all forms of brain damage such as dementia or vascular disease (Glosser et al., 1977; Cukierman et al., 2005; Rapp and Reischies, 2005; Rosano et al., 2008). Performance on various digit symbol matching tests has been consistently linked with obesity and diabetes (Brands et al., 2005; Cukierman et al., 2005; Cournot et al., 2006; Palta et al., 2014). Due to its repeatability and sensitivity to state variables, this test can be administered many times in high frequency measurement designs or administered at single time points, and it could serve as an outcome of interventions.

Individually or in combination, the two GCI tests provide a means of understanding trait and state-related neuropsychological functioning. Taken together, these measures can be thought of as analogous to body mass index (BMI), which uses height (stable) and weight (variable) to capture an important aspect of health that varies across individuals. Insofar as the matrix reasoning scores can reflect “premorbid” or stable aspects of cognitive performance, the digit symbol matching scores can be interpreted with respect to matrix reasoning scores to identify discrepancies (e.g., abnormally slow processing speed given high non-verbal reasoning capability) that could be indicators of poorer health (Schoenberg et al., 2003; Ball et al., 2007; Lezak et al., 2012). However, the degree to which matrix reasoning scores are themselves sensitive to brain health can differ across conditions (Ryan et al., 2005).

### Test Versions, Citations, and Weblinks

The GCI can be administered using most digital devices like laptops, desktops, tablets, and smartphones and have existing large datasets that can be used for normative comparisons. These tests are available open source and adapted from standard format matrix reasoning items and also include item-level statistics that can be used for interpreting test scores and building shorter test versions. Please email testmybrain@gmail.com for access to a free study page with digital test versions for research use.

TestMyBrain.org Matrix Reasoning Test (DEMO LINK)
https://www.testmybrain.org/tests/matrix_discontinuerule/index_numbered.html.
TestMyBrain.org Digit Symbol Matching (DEMO LINK)
http://www.testmybrain.org/tests/DigSymbCoding/DSC.html.

### Scoring and Primary Outcome Variables

#### Visual Pattern Reasoning (Matrix Reasoning Format)

The primary outcome variable for this test is accuracy across 35 test trials, which reflects general cognitive or non-verbal reasoning ability. Reaction times can also be used as a secondary measure but are most useful for identifying invalid data, such as trials on which the participant was not paying close attention to their responses. For the test link provided, accuracy is automatically calculated.

#### Digit Symbol Matching Speed (Digit Symbol Substitution Format)

The primary outcome variable for this test is throughput, or number of correctly matched symbols, within the administration time (90 s), which reflects processing speed. While the accuracy is not used as an outcome measure, it provides important validity information. For example, poor accuracy, defined as <75% correct or less than 50% correct depending on whether the target population is healthy or impaired, can indicate the participant was not paying close attention and therefore the number of correct responses cannot be interpreted. For the test link provided, number of correctly matched symbols (throughput) is automatically calculated.

## Standard Battery (∼30 Min)

### Purpose

The Standard Battery (see Table 3) is constructed to be short (∼30 min total, including the GCI) and easy to use while also enabling measurement of the domains likely to be the best predictors, mediators, and/or moderators of outcomes in adult obesity and diabetes trials. It includes the GCI and four other tasks that assess executive function and cognitive control, learning and memory, and decision-making. The Extended Battery includes tasks that cover the remaining cognitive domains.

### Executive Function/Cognitive Control

The tasks selected for the Standard Battery measure two constructs of executive function and cognitive control that are relevant to obesity and/or diabetes: response inhibition and task switching. We used the factor structure of executive function developed by Miyake et al. (2000), which categorizes executive function into 3 sub-domains: inhibition, working memory, and switching. Each of those three domains have been consistently related to obesity in the past (Vainik et al., 2013; Emery and Levine, 2017; Yang et al., 2018). However, working memory is strongly related to general intelligence (Eisenberg et al., 2019) measured by the GCI, which is why we decided to focus on inhibition and switching here. The selected tasks have high test–retest reliability, predictive power, and are available in open source formats. Additionally, the tasks measuring response inhibition and higher-order planning are face valid.

The CoreNP expert group selected the go/no-go task as a measure of response inhibition. This task involves presentation of a single stimulus on each trial and the participant is instructed to respond if the stimulus falls into a particular class (the “go” stimulus) but not to respond if it falls into another class (the “no go” stimulus). For example, a participant might respond to “*X*” but not to “*O*.” When the no-go condition occurs relatively infrequently (e.g., less than 1/3 of trials), success on the task is thought to require inhibition of the prepotent “go” response. The task should include sufficiently more go than no-go trials, use rare no-go trials, and short trial durations (Wessel, 2018). In the literature, d’ is the most common outcome measure reported; however, the commission error rate (i.e., the number of responses on no-go trials) may be a more direct measure of response inhibition.

Task switching is a major component in cognitive flexibility, and relationships between switching and obesity have been established with several tasks, such as Trail-Making Test B and the Wisconsin Card Sort Task (Vainik et al., 2013; Emery and Levine, 2017; Yang et al., 2018). We selected the Dimensional Change Card Sort (DCCS) task (Zelazo, 2006). This task involves presentation of a stimulus that varies along two dimensions, color and shape, and the participant is instructed to respond to only one of the dimensions on each trial. The rules governing responses change occasionally, and the primary measure is the number of correct responses. We proposed the use of DCCS, as it is available through the NIH toolbox and has also been linked to obesity (e.g., Vainik et al., 2018).

### Learning/Memory

While there is not strong evidence for an association between memory and obesity in otherwise healthy young individuals, such an association appears to emerge with age. A higher BMI in middle age is associated with an accelerated age-related decline in memory ability (Cournot et al., 2006) and later dementia (Whitmer et al., 2005). Being overweight has been associated with a reduced hippocampal volume in otherwise healthy participants aged 60–64 years (Cherbuin et al., 2015). This effect is even more apparent in older adults who are obese, and this relationship is present even when cardiovascular and blood glucose factors are taken into account (Raji et al., 2010). Obesity might therefore accentuate other factors that compromise memory performance such as normal aging or age-related neurodegenerative disease. Tasks that are highly sensitive to hippocampal function and that require episodic or relational memory or that involve arbitrary associations are likely to be the most sensitive. Learning and memory appear to be more useful as outcome measures, reflecting the sensitivity of the hippocampal system to metabolic factors (Cheke et al., 2016; Hargrave et al., 2016; Higgs and Spetter, 2018).

The Picture-Sequence Memory task from the NIH Toolbox was selected given its psychometric properties, availability, and short duration. In this task, participants study sequences of pictures of objects and actions, and then must recall the order in which the items appeared. Hayden et al. (2018) used this task to assess cognition after an intensive lifestyle weight loss intervention in participants aged 45–76 years with type II diabetes. The intervention had no effect on the Picture-Sequence Memory task across participants, but a subset of participants with the highest BMI had decreased performance after the intervention, suggesting that significant weight loss might not have beneficial effects on memory in older participants (Hayden et al., 2018). Additional research is needed to determine the suitability of the Picture-Sequence memory task. There is one study suggesting that a verbal learning task could be sensitive to a western diet manipulation (Stevenson et al., 2020), but this evidence is not conclusive and demonstrates a need for further research in this area.

### Decision-Making

The Kirby Delay Discounting task was selected because temporal (delay) discounting has been widely studied in relation to obesity and overweight status and other impulsivity-related clinical conditions (Vainik et al., 2013, 2018; Volkow and Baler, 2015; Barlow et al., 2016; Yang et al., 2018). The discount factor k, or the rate at which people discount future rewards, can measured accurately with very brief tasks (Koffarnus et al., 2015). The primary outcome of a delay discounting task is the discount rate, with larger values of *k* indicating increased impatience and preference for rewards that are available immediately. Cross-sectional evidence suggests that delay discounting is steeper in individuals with overweight or obesity compared to healthy-weight groups, with small-to-moderate effect sizes. Though delay discounting variables are considered potential predictors or moderators of behavioral or health outcomes; to our knowledge, there are only limited data on delay discounting as an outcome in adult obesity and diabetes trials (Stein et al., 2017).

## Extended Battery (∼75 Min)

The extended battery includes the GCI, Standard Battery, and additional tasks below. The tasks are also summarized in Table 4.

**TABLE 4:** Extended battery.

**Test**	**Version/source**	**Administration time**	**Scoring/primary outcome variables**
**Reward/motivation**			
Effort expenditure for rewards task (EFfRT)	Treadway et al., 2012	15 min	Global: overall percentage of high effort choices. Probability effects: variation in percentage of high effort choices as a function of probability of receiving the outcome if task completed. Reward effects: variation in percentage of high effort choices as a function of reward magnitude.
Game of dice	Brand et al., 2005	10 min	Percentage of times an individual chooses a riskier option over a less risky option.
**Attention/working memory**			
Digit span working memory	WAIS-IV: https://www.pearsonassessments.com	5 min	Total score across all three conditions (forward, backward, and sequencing) as well as separate forward, backward, and sequencing scores.
Dot pattern expectancy(DPX)	NIMH-CNTRICS/CNTRACS http://cntracs.ucdavis.edu/tasks	10 min	Overall score is discriminability (*d*′) in the AX condition, but for examination of WM it is more informative to examine BX errors and possibly AY errors.
Visual search	Gold et al., 2007, 2018 www.scienceofbehaviorchange.org (in process)	5–10 min	Search efficiency; quantified as the slope of the function relating reaction time to set size.
**Learning/memory**			
Spatial reconstruction/I-position	Watson et al., 2013	15 min	Accuracy
**Sensation/perception**			
Heartbeat awareness	Schandry, 1981	6 min	Accuracy
**Decision-making**			
Holt–Laury risk taking for monetary outcomes	https://scienceofbehaviorchange.org/measures/holt-laury-risk-titrator/	3 min	Number of risky choices (*n*/10)
5-Trial adjusting delay discounting*	https://scienceofbehaviorchange.org/measures/five-trial-adjusting-delay-discounting-task/	27 s	Discount factor, *k*

*This shortened discount task might be used as an alternative to the 5 min, 25 question Kirby Delay Discounting task recommended in the Standard battery.

### Reward/Motivation

The tasks chosen measure constructs of reward-seeking and motivational processes that are relevant to obesity and diabetes including the willingness to exert effort to acquire rewards and risk-taking (a shared construct with the decision-making domain). The CoreNP expert group defined reward seeking as the experiences that accompany receiving rewarding outcomes like money, food, positive feedback, etc. or failing to receive rewarding outcomes. This definition also includes Pavlovian and operant learning about cues that predict rewarding outcomes from experiences of receiving or not receiving such outcomes, determining how much effort is required to pursue rewards, and making choices that balance the risks of winning versus losing rewarding outcomes.

The performance on the effort expenditure for reward (EFfRT) task was chosen because it is a valid measure of effort allocation toward reward, has high test–retest reliability, is open-source, and tolerable (Reddy et al., 2015). This task has been linked to adherence to weight loss interventions (Mata et al., 2017). The EFfRT asks participants to choose between an easy task (21 button presses with dominant index finger) for $1 and a hard task (100 button presses with non-dominant pinky finger) for varying amounts of money (>$1). The amount of money a participant can earn for the hard task varies across trials ($1.60–$4.00+), as does the likelihood that they will actually be awarded the money should they successfully complete the tasks. The likelihood typically ranges between 50 and 88%, though some versions of the task also use a 12% condition.

The “Game of Dice” task was chosen as a measure of risk-taking because it is associated with decision-making processes involved in long-term weight loss (Brockmeyer et al., 2017). This task is a valid measure of risk behavior (Brand et al., 2005), has high test–retest reliability (Starcke et al., 2017), and is tolerable to participants. Participants are told they should try to obtain as much money as possible by predicting the outcome of a die roll. They are shown a screen and asked to predict which of two choices they think will contain the number that will appear on the die. The choices are between one specific single number or a series of 2–4 numbers. Choices with fewer number options are “riskier.” For example, choosing a single number is associated with a 1/6 win likelihood, with the outcome being ±$1,000. In contrast, choosing a string of 4 numbers is associated with a 4/6 win likelihood, but with an outcome of ±$100.

### Attention/Working Memory

The tasks chosen are measures of sustained attention and working memory chosen are sensitive indicators of cognitive impairment, can be given in a relatively short amount of time, and can be flexible given the needs of the study. Both attention and working memory are involved in setting goals and decision making, which in the context of obesity and diabetes can include decisions on what to eat, when and how much to exercise, when and how much to sleep, and self-monitoring diet, physical activity and insulin. Digit Span is one of the most well used measures of attention and working memory. While Digit Span was once referred to as a test of attention, it has more recently been considered a measure of working memory or auditory working memory based on factor analytic studies (Holdnack et al., 2011). Digit Span has been associated with BMI (Elias et al., 2003; Di Blasio et al., 2010), in particular Digit Span forward has been negatively associated with BMI (Gunstad et al., 2007, 2010). Digit Span has shown differentiation between individuals with overweight or obesity and those who are a healthy weight (Elias et al., 2003; Coppin et al., 2014) and with binge eating disorder (Duchesne et al., 2010). Digit Span scores seem to improve with weight loss (Halyburton et al., 2007; Brinkworth et al., 2009). As in all research, there are a number of studies that show a lack of association between digit span and overweight and obesity (Strachan et al., 1997; Awad et al., 2004). However, it is important to consider additional factors, such as insulin sensitivity (Gonzales et al., 2010) and physical activity (Brown et al., 2012; Basso et al., 2015; Huang et al., 2018) which could be potential moderators among BMI, attention and working memory. The Dot Pattern Expectancy (DPX) and visual search tasks were chosen to measure the constructs of attention and working memory with relevance to obesity and/or diabetes. While there is a paucity of evidence supporting their role in obesity and/or diabetes, specifically, the other strengths of these tasks and the relationships to other important health behavior outcomes led the group to recommend these tasks. Both attention and working memory are involved in setting goals and decision-making, which in the context of obesity and diabetes can include decisions about what to eat, when and how much to exercise, when and how much to sleep, and the self-monitoring of diet, physical activity, and prescribed treatment. Further, the DPX task can also capture tendencies toward proactive or reactive control modes (Braver, 2012). Therefore, the task may also be useful to understand executive function.

The DPX task, which measures sustained attention and working memory, was selected by the NIMH Research Domains Criteria (RDoC) initiative expert group and the NIMH Cognitive Neuroscience Treatment Research to Improve Cognition in Schizophrenia (CNTRICS) group for assessment of working memory in psychotic disorders following analysis of its psychometric properties, feasibility, and validity data in schizophrenia and other psychotic disorders (see Barch et al., 2008; MacDonald, 2008; Lopez-Garcia et al., 2016). It was carried forward and further developed by the Cognitive Neuroscience Test Reliability and Clinical Applications for Schizophrenia (CNTRACs) Consortium. The task was coded for implementation using the E-Prime software package and is freely available on the CNTRACS website (see Table 4).

The visual search task measures selective attention while minimizing working memory and executive function requirements (Gold et al., 2007). We recommend a version of conjunction visual search implemented by Gold et al. (2007, 2018) that requires the shifting of covert attention between items to find the target. Participants are presented with a search array comprising a variable number of items (set size). The items are horizontal or vertical ellipses, which can be red or blue. Participants search for a target item defined by a conjunction of orientation and color (e.g., red + vertical), and report whether or not the target ellipse has a gap. The primary measure of visual search is reaction time (RT); however, the more important measure is the slope of the RT x set size function, which serves as an index of search efficiency. Accuracy is a secondary dependent measure. Search efficiency has not been studied as an outcome measure in obesity/diabetes.

### Learning/Memory

In addition to the Picture-Sequence Memory task in the Standard Battery, the CoreNP expert group recommends the Spatial Reconstruction Test, which is relatively brief and has also been shown to be sensitive to hippocampal involvement (Watson et al., 2013). In this task, participants study an array of shapes on the screen in each trial. The shapes disappear, and after a short delay they appear at the top of the screen and the participant must drag each shape to the location in which it had appeared. In a recent study, performance on this task in overweight individuals was related to serum lutein (Cannavale et al., 2019). Because lutein is important for brain function and is reduced in individuals with obesity, these results suggest that this consequence of obesity may result in impaired hippocampal-dependent memory.

### Sensation/Perception

Sensation refers to the process of realizing internal and external environments through touch, taste, sight, sound, smell, and interoception. Perception refers to the ways in which these signals are interpreted. The CoreNP expert group evaluated the evidence for cross-sectional and predictive associations among BMI and eight domains of sensation and perception that included pain, taste, smell, body schema, interoception, and visuospatial perception. The mechanisms linking these domains to outcomes of obesity and diabetes trials were also considered, as were practical considerations such as cost and burden to the participant and investigators.

For the Extended Battery, we decided to measure interoceptive accuracy with the Schandry task (Schandry, 1981). Levels of interoceptive cardiac accuracy are low in type 2 diabetes (Grammes et al., 2018). This task is the most widely used measure of cardiac interoception due to its simplicity, assessment speed (6 min), and predictive validity for key variables of interest. It uses pulse-oximetry, a common and widely used medical tool. The Schandry task provides a measure of cardiac interoceptive accuracy by comparing how many heartbeats participants count to how many are measured by the pulse oximeter. This task was selected because performance is impaired in obese individuals, and a greater BMI is associated with impaired performance accuracy (Herbert et al., 2013; Herbert and Pollatos, 2014; Koch and Pollatos, 2014). Performance on the Schandry task is also related to gastric interoception (Van Dyck et al., 2016) and thus it offers a fast and efficient way to detect general visceral sensitivity.

### Decision-Making

In addition to delay discounting, the CoreNP expert group recommends the assessment of risk attitude with the Holt–Laury Risk Taking for Monetary Outcomes. While less-studied in obesity and diabetes, risk attitude (or risk preference), is conceptually and behaviorally related to temporal discounting. The term “risk” has many meanings that have been operationalized in different ways; we refer here to a formal economic notion of variability in the outcomes of monetary prospects. Like delay discounting, risk can be measured accurately with very brief tasks (Koffarnus et al., 2015). Risk variables are considered potential predictors or moderators of behavioral or health outcomes; to our knowledge, they have not been studied as outcomes.

The Holt–Laury task involves a series of decisions between 10 pairs of hypothetical lotteries, with more risky outcomes (e.g., 10% chance of winning $190, 90% chance of $5) vs. safer outcomes (e.g., 10% chance of winning $100, 90% chance of winning $80). The number of pairs for which the participant chooses the risky option (*n*/10 choices) gives a measure of risk attitude (with larger *n* indicating risk seeking and smaller n indicating risk averse preferences).

## Next Steps

There were two overarching purposes of the initiative: (1) to identify the highest priority cognitive domains for obesity and diabetes research and clinical trials based on the current evidence and (2) to provide researchers with suggested measures within each domain, identifying specific measures that are valid and available in the public domain wherever possible. This publication represents the culmination of this first step for the NIH Core Neuropsychological Measures for Obesity and Diabetes Project. We will continue to evaluate specific measures, and additional recommendations or modifications will be updated in this paper as appropriate.

While the expert group has suggested strategies for implementing and disseminating these measures and batteries into studies, the workshop did not focus on implementation and dissemination. Challenges in using these measures and batteries were not considered or discussed in depth. For example, it is likely that burden to the researcher and participant in access and use of these measures could limit broad adoption of these batteries into adult obesity and diabetes studies. Future efforts should address these and other challenges to promote use of the best neuropsychological measures to advance obesity and diabetes research.

Additional data collection considerations and analysis tools are provided in Appendices B,E. Another exhaustive resource for important covariates is the Accumulating Data to Optimally Predict obesity Treatment (ADOPT) Core Measures Project. The project outlines factors from behavioral, biological, environmental, and psychosocial domains (MacLean et al., 2018).

## Implementation and Dissemination

### PHASE I: Initial Dissemination of the General Cognitive Index

To begin, we recommend communicating widely the importance of generating a neuropsychological profile of obesity and diabetes while simultaneously encouraging researchers engaged in upcoming and ongoing clinical trials to incorporate the General Cognitive Index (GCI) into their experimental designs. The two tests included in the GCI can be thought of as analogous to the height and weight measures researchers currently acquire to calculate the BMI. While matrix reasoning scores are expected to remain stable (like height), and provide an estimate of premorbid functioning, digit symbol matching scores are expected to be more sensitive to changes in brain health. The GCI can be easily acquired at no cost through TestMyBrain.org (email: testmybrain@gmail.com). We anticipate Phase I would last approximately 6–18 months.

### PHASE II: Initial Dissemination of Standard Battery and Extended Battery

We next recommend researchers engaged in upcoming and ongoing clinical trials on obesity and/or diabetes include the Standard or Extended Battery in their experimental designs. Finalized versions of the Standard Battery and Extended Battery might require a meeting of domain experts to agree on exact test parameters and recommended versions. It is important that there are inexpensive and easily accessible test options and at least one test option that can support a large-scale study requiring formal data management and software infrastructure. A small psychometric study, similar to MATRICS, could be helpful in identifying comparability between test versions and any weaknesses in particular test formats. It would also be helpful to identify, support, and/or develop infrastructure for aggregating data. We anticipate Phase II would last approximately 6–24 months.

### PHASE III: Evaluation of Initial Findings and Further Refinement of Measures

We intend for the Standard and Extended Batteries to be evaluated and refined as needed. To this end, we recommend either a formal survey of grantees and investigators using the recommended batteries or the GCI, or that the batteries be incorporated into ongoing studies to rapidly evaluate the predictive validity and psychometrics of the recommended tests. Any potential issues with sociocultural biases and accessibility across groups should be evaluated and addressed. We also recommend support for trainees to write reviews or papers aggregating the results from the recommended tests across studies. Formal recommendations should be created for data sharing of all the neuropsychological battery data elements and common data models and for best practices around the return of research results. After evaluation, we recommend that tests showing no early evidence of utility be eliminated or replaced. We anticipate Phase III would last approximately 18–36 months.

### PHASE IV: Final Implementation

We recommend the development of infrastructure to support the final batteries. Such infrastructure might rely partly or entirely on existing infrastructure, like NIH Toolbox. We also recommend the development of publicly available test norms, documentation, scoring procedures, and training protocols to support the use of the batteries as experimental tools in clinical trials.

When finalized, we recommend that the Core Neuropsychological Measures for Obesity and Diabetes Trials Project batteries use standardized text for grants, method sections in publications, Institutional Review Board proposals, online training, tutorials; and digital identifiers, where possible.

## Knowledge Gaps and Research Opportunities

Our understanding of the best neuropsychological measures to predict responses in obesity and diabetes trials is still early, and the data are somewhat limited. Additional research is needed to validate the proposed measures and determine which are the most valuable predictors, mediators, and moderators of clinical outcome. Thus, as described in Phase III above, the proposed measures will undoubtedly be refined as they are incorporated into ongoing and new studies. Though the recommended batteries may be modified based on new results, they cannot be evaluated until they are incorporated into clinical obesity and diabetes trials. Based on existing results from neuropsychological studies of obesity and diabetes, we believe that the proposed cognitive tests are the best place to start.

There were a number of important research gaps identified. First, much of the existing research has focused on BMI and/or obesity with more limited research in insulin resistance and diabetes or in the contributions of diet, physical activity, sedentary behavior, diet, and/or caloric intake to cognitive functioning. Also, the task-BMI evidence was sometimes based on one-off studies that need replicating. While there is some evidence that performance on proposed tasks relates to treatment adherence or long term outcomes, we know little about whether variation in these constructs or performance on these tasks predict, e.g., BMI, insulin sensitivity, and/or glycemic variability across the lifespan, or the development of obesity and diabetes. Though the initial work on test–retest reliability is promising, some tasks in the extended battery had less than optimal reliability (*r* < 0.7), so future psychometric work may be needed to optimize those tasks or replace them with more stable tasks. Alternatively, the number of measurements per participant could be increased to improve the signal-to-noise ratio (Sliwinski et al., 2018). More work is also needed to ensure that tasks are well suited as predictors, mediators, and/or moderators of outcomes in observational and treatment trials. There is a particular need to test these measures as predictors of outcomes as there has been very limited, rigorous research in this area. In addition, change in cognitive performance could also be an outcome of obesity. For instance, bariatric surgery is known to improve performance in multiple cognitive domains (Thiara et al., 2017). Building an evidence base to identify which cognitive domains are predictors, moderators, and/or outcomes for obesity and diabetes trials is a clear need.

Some of the recommended tasks use monetary outcomes. While performance on these tasks has been shown to be related to obesity and BMI and to a lesser extent, diabetes, it is important to directly compare versions that use primary rewards (food, drink, etc.) versus secondary rewards (i.e., money) to determine if there is greater predictive utility for one type of reward versus another.

At this time, food-specific tasks have not been sufficiently validated or standardized to be recommended. However, such tasks can be briefly and validly measured and have some evidence of relevance in obesity, diabetes, and other aspects of health-related behaviors. There is also some evidence that such domain-specific approaches might have larger effect sizes than tasks using monetary outcomes (Wu et al., 2016). Existing tasks that use food cues or require decision-making in the obesity and diabetes context have high ecological validity, but are not standardized and would require careful consideration of socio-cultural influences and tailoring to meet assessment objectives.

Like many areas of physical and mental health, the roots of behaviors and developmental trajectories associated with obesity and diabetes emerge early in the course of development. As such, it has become increasingly clear that early detection and identification are critical. To effectively conduct work on early detection, identification, and prevention, we need to facilitate the use of paradigms and measures that can either be used in young populations or facilitate the translation of such measures for use in children, including very young children. This will be a challenge, as the majority of the measures described above have been validated in adults but have rarely been used in younger populations. Therefore, creative modifications of the same paradigms will be needed for use in younger children, along with appropriate validation studies. In addition, we will need to consider whether there are alternative ways to obtain measures of the same constructs in very young children, such as parent report or observational measures.

Finally, obesity and diabetes are diseases that are overrepresented in certain racial and ethnic groups. Unfortunately, many neuropsychological measures have inadequate or unknown performance properties in these groups (Manly, 2008; Henrich et al., 2010). We have tried to select tasks that are known to perform well (or should) in culturally and linguistically diverse individuals; however, there are still known limitations, even with these tasks. Therefore, more work is necessary to develop, adapt, and test the performance of tasks that do an adequate job of assessing the neuropsychological function of the culturally and linguistically diverse individuals that will be participants in our clinical studies and the recipients of our therapies (Barrett, 2020). A statement should be included as to whether task administration is appropriate for a given racial/ethnic and/or immigrant group.

## Author Contributions

KD’A, CS, DS, UV, and LS led the writing group and contributed substantially to coordination and preparation, writing and editing, and revision of the manuscript. All authors contributed to the article and approved the submitted version.

## Conflict of Interest

The authors declare that the research was conducted in the absence of any commercial or financial relationships that could be construed as a potential conflict of interest.

## Footnotes

1 www.nihtoolbox.org

2 https://memory.ucsf.edu/research-trials/professional/examiner

3 https://www.pearsonassessments.com/
