# Corrigendum: Core Neuropsychological Measures for Obesity and Diabetes Trials: Initial Report

**DOI:** 10.3389/fpsyg.2020.612441

**Published:** 2020-11-09

**Authors:** Kimberlee D'Ardenne, Cary R. Savage, Dana Small, Uku Vainik, Luke E. Stoeckel

**Affiliations:** ^1^Department of Psychology, Arizona State University, Tempe, AZ, United States; ^2^Department of Psychology, University of Nebraska–Lincoln, Lincoln, NE, United States; ^3^Center for Brain, Biology and Behavior, Department of Psychology, University of Nebraska–Lincoln, Lincoln, NE, United States; ^4^Modern Diet and Physiology Research Center (MDPRC), Department of Psychiatry, Yale School of Medicine, Yale University, New Haven, CT, United States; ^5^Department of Psychology, Yale University, New Haven, CT, United States; ^6^Institute for Diabetes Research and Metabolic Diseases of the Helmholtz Center Munich at the University of Tübingen, Tübingen, Germany; ^7^Institute of Psychology, Faculty of Social Sciences, University of Tartu, Tartu, Estonia; ^8^Department of Neurology and Neurosurgery, Faculty of Medicine, McGill University, Montreal, QC, Canada; ^9^National Institute of Diabetes and Digestive and Kidney Diseases, National Institutes of Health, Bethesda, MD, United States

**Keywords:** executive function, cognitive control, reward, motivation, decision-making, memory, sensation, perception

In the original article, there was an error. Information at the end of the Acknowledgments section was inadvertently omitted.

A correction has been made to Acknowledgments to read as follows:

We would like to thank the executive committee members for assistance with organization and planning the activities that led to this report and the executive committee and workshop participants for contributing content expertise, writing, and edits to the report (see Supplementary Appendix C for listed names and affiliations). We would also like to thank Drs. Patrick Bissett, Desiree Byrd, Xavier Cagigas, Laura Holsen, and Kristin Javaras for their review and insightful feedback on the preprint (i.e., *NutriXiv*) version 1 of the report. We also thank Richard Gershon and Molly Wagster who gave excellent input on development of the NIH Toolbox, which was helpful in crafting some of the thinking and language that ended up in the report. We would further like to acknowledge the writing contributions for the content of each section came from members of the project executive committee and workshop participants. The content is solely the responsibility of the authors and does not represent the official views of the NIH or federal government.

The authors apologize for this error and state that this does not change the scientific conclusions of the article in any way. The original article has been updated.

