# Supplementary material for: Core Neuropsychological Measures for Obesity and Diabetes Trials: Initial Report
Source: Front Psychol. 2020 Sep 24;11:554127. doi: 10.3389/fpsyg.2020.554127 (PMC7557362; doi:10.3389/fpsyg.2020.554127)
Supplement: Supplementary file 1 [file Data_Sheet_1.PDF]

Supplementary Material

Supplementary material for:

d'Ardenne, K., Savage, C., Small, D., Vainik, U., & Stoeckel, L. E. (2020). *Core Neuropsychological Measures for Obesity and Diabetes Trials: Initial Report*. https://doi.org/10.31232/osf.io/7jygx

- Appendix A: Psychometric Considerations
- Appendix B: Other Data Collection Considerations
- Appendix C: Workshop Organizational Structure
    - Chairs
    - Executive Committee
    - Working groups
- Appendix D: Tasks Not Recommended
- Appendix E: Test-Retest Reliabilities and Power Calculation Tool

Supplementary Material

APPENDIX A: PSYCHOMETRIC CONSIDERATIONS

HIGH PRIORITY

1. How strong is the evidence that the task provides a valid measure of the indicated construct?
2. Does the task have strong test-retest reliability?
3. Does the task have good predictive validity, especially for obesity, diabetes, or other consequential health or real world outcomes? For what types of studies (e.g., lab-based, observational, mechanistic and/or interventional clinical trials, mobile/web assessments)?
4. How long does the task take to administer (including any necessary instructions, set-up, and practice) and how tolerable and/or burdensome is the task for participants?
5. Is the task open source or available for open source?
6. Can it be used across different cultural or sociodemographic groups?

MEDIUM PRIORITY

1. To what extent is the task (or different versions of the task) suitable for use across laboratory-based studies, clinical trials (as a measure of target engagement or clinical outcome), and/or high-throughput screening settings?
2. To what extent is the task suitable for administration on the web or using mobile devices, in a participant's home or other naturalistic environment, and with the participant's own device or other readily available device?
3. Are adequate normative data available across age, gender, education, ethnicity, and socioeconomic status?
4. Is the task widely used currently or has its use been limited to a few research groups?
5. Is the task sensitive to within-person change?
6. Are the relationships between task performance and clinical feature(s) relevant to obesity and diabetes known?

LOW PRIORITY

1. Are parameters for administering the task (e.g., number of trials, stimulus characteristics, and primary dependent measure) standardized on an empirical basis?
2. Can the task be used (or adapted for use) with children, older adults, and other special populations?
3. Can the task (or its analog) be used in non-human animals? Is a non-human animal version available?
4. Are alternate forms available?
5. Is the relationship between task performance and neural signal known or is the task adaptable for the neuroimaging environment

Workgroup members are asked to bear the following considerations in mind when choosing tasks:

a. When choosing among measures, a task that relates to clinical features (particularly functional status) is preferred. Workgroup members should consider that some measures are influenced by culture or sociodemographic characteristics. Workgroup members should consider the acceptability of tasks to participants. Some might be too difficult or burdensome and that perceived difficulty or burden may vary across population groups.
b. When nominating a task, the workgroup should note where possible:

i. the particular psychometric properties of the task or paradigm (where information is available) and the subpopulations that have been tested;
ii. whether the task measures a state or trait;
iii. the appropriate use of the task (e.g., whether it is suitable for longitudinal research versus single administration);
iv. whether the parameters for administering a task (e.g., number of trials, stimulus characteristics, and primary dependent measures) have been standardized based on empirical evidence;
v. variation in the parameters and length of task needed to obtain the desired level of sensitivity across populations; and
vi. the settings in which the task can be used (e.g., laboratory only, clinical trials, mobile or web assessment)

c. For each task, workgroups will be asked to give:
    i. Task Name (name, version, citations)
    ii. Construct Measured
    iii. Administration Time (min/sec)

Supplementary Material

APPENDIX B: ADDITIONAL DATA COLLECTION CONSIDERATIONS

The Core Neuropsychological Measures for Obesity and Diabetes Trials are a restricted set of neuropsychological measures that we recommend be included in clinical trials of obesity and/or diabetes. The focus of the CoreNP workgroup was to identify valid measures of cognitive and perceptual functioning; other data relevant to obesity and/or diabetes clinical trials were outside the scope of this project. The addition of other types of measures and data could add explanatory power to studies using the Core NP measures. We further describe data collection considerations, such as the importance of test-retest reliability, study design, and power analysis.

Personality questionnaires can support neuropsychological measures, as they capture wide aspects of obesity-related behaviors, thoughts and feelings. Many personality facets have small-but-consistent associations with concurrent BMI (Vainik, Dagher, et al., 2019) and BMI change (Sutin et al., 2011). The small effects (|r|>0.07) can be aggregated into personality "risk" scores (r=0.15, Vainik, Dagher, et al., 2019). Personality-obesity effects are stronger for eating-specific questionnaires (r=0.30, Vainik et al., 2015). Despite different names, many eating-related questionnaires measure the same uncontrolled eating which has bidirectional longitudinal associations with BMI (Vainik, García-García, & Dagher, 2019).

Obesity is also known to be associated with hundreds of genes, which have small incremental associations with obesity (Yengo et al., 2018). If genotype data are collected, a polygenic risk score for obesity can predict BMI (r=0.24 Yengo et al., 2018). Using this polygenic score as a covariate in RCTs can reduce sample size requirements by ~7% (see Excel power calculation tool in Appendix E and supplementary material Ch 8 in Rietveld et al., 2013) due to increased variance explained. However, the utility of polygenic scores will be lower for non-Caucasian samples. Considerations about these expanded data are addressed in the Accumulating Data to Optimally Predict Obesity Treatment (ADOPT) Measures: https://www.ncbi.nlm.nih.gov/pubmed/29575780. The ADOPT recommendations examined several domains, including behavioral, psychosocial, and biological domains.

Many of these measures overlap with existing or planned neuropsychological batteries in other areas of science. For example, the MyCog (https://www.detectcid.org/northwestern) is a brief battery being developed for studies of cognitive impairment and dementia that overlaps with part of the Core NP standard battery (NIH Toolbox Pic Memory and Dimensional Card Change tasks). Overlap with other batteries across other studies, with different research questions and/or disease focus, will allow for data comparison to address many additional research questions beyond what might be addressed in obesity and/or diabetes studies alone.

Several other potential issues exist. One of them is test versioning. Digital cognitive tests are subject to changes. We suggest documenting the version and time/date when the version of the test used was created. Similarly, when the tests are adapted for international use, care needs to be taken that international versions are comparable with US versions. We suggest reporting means and standard deviations of both normed and raw scores, so that maximum comparability between different studies is possible. These and other challenges for digital neuropsychology have lately been summarised by Germine et al (2019).

Current report has stressed the importance of test-retest reliability. An effect must have high test-retest reliability to serve as a valid predictor, because the relationship among variables is limited by their reliability. High test-retest reliability for an effect requires that the measures exhibit substantial

variability across individuals. Many effects tested in cognitive psychology show low test-retest reliability exactly because they vary little across individuals (Hedge et al., 2017), and they subsequently show little predictive ability for real-world outcomes (Eisenberg et al., 2018). The test-retest reliabilities of tasks listed in current report are listed in Appendix E.

Besides selecting measures, it will be critical to give careful consideration to study design. Relevant factors include test-taking support (e.g., native language version, language translation, interpreter for translation to native language (if native language version is not available, vision/hearing/motor support/accommodation, etc.), task administration details (e.g., device used, order of task administration, time of day, participant-rated fatigue, incentives provided for task performance), other circumstances that may impact performance (e.g., medical/psychosocial issues – either chronic or acute, substance use, sleep quantity/quality).

Similarly, one should estimate necessary sample sizes and expected effect sizes for appropriately powered studies. For example, in a between-person, cross-sectional design, predicting adult BMI from childhood IQ has an effect size of d=0.18-0.12/r=0.09-0.06 (Chandola et al., 2006; Chinn, 2000); this effect requires 1,000-2,200 participants to be detected with 80% power and p=0.05 (Champely et al., 2018). However, using a repeated-measures (longitudinal) design, assuming test-retest reliability *r*=0.6 for a given measure, an effect size *r*=.06 could be detected with a sample size of 440 participants. It is also reasonable to expect interventions might have stronger effects detectable with smaller sample sizes. A typical effect size in psychology is *r*=0.2, which needs 193 participants in a cross-sectional, between-person design, but only 39 participants in a longitudinal, within-person design (https://daniellakens.blogspot.com/2016/11/why-within-subject-designs-require-less.html). However, note that within-person designs are vulnerable to regression to the mean (Barnet et al., 2005) – that is more extreme scores in cognition measures may naturally normalize over time. Therefore, a control group or appropriate statistical tools are necessary to counter regression to the mean (Barnet et al., 2005). Brysbaert (2019) provides another excellent resource for designing well-powered experiments, including discussion of power considerations with accessible language and pre-calculated sample size tables for common research designs. More information about test-retest reliability for the recommended tasks in this report and an Excel power calculation tool can be found in Appendix E.

Additional references in Appendix B:

Barnett, A. G., van der Pols, J. C., & Dobson, A. J. (2005). Regression to the mean: What it is and how to deal with it. International Journal of Epidemiology, 34(1), 215–220.

Brysbaert, M. (2019). How many participants do we have to include in properly powered experiments? A tutorial of power analysis with reference tables. Journal of Cognition, 2(1), 16.

Champely, S., Ekstrom, C., Dalgaard, P., Gill, J., Weibelzahl, S., Anandkumar, A., … Rosario, H. D. (2018). Pwr.r.test function | R Documentation (Version 1.2-2). Retrieved from https://web.archive.org/web/20190528073728/https://www.rdocumentation.org/packages/pwr/versions/1.2-2/topics/pwr.r.test

Chandola, T., Deary, I. J., Blane, D., & Batty, G. D. (2006). Childhood IQ in relation to obesity and weight gain in adult life: The National Child Development (1958) Study. International Journal of Obesity (2005), 30(9), 1422–1432.

Chinn, S. (2000). A simple method for converting an odds ratio to effect size for use in meta-analysis. Statistics in Medicine, 19(22), 3127–3131.

Eisenberg, I. W., Bissett, P. G., Zeynep Enkavi, A., Li, J., MacKinnon, D. P., Marsch, L. A., & Poldrack, R. A. (2019). Uncovering the structure of self-regulation through data-driven ontology discovery. *Nature Communications*, *10*.

Germine, L., Reinecke, K., & Chaytor, N. S. (2019). Digital neuropsychology: Challenges and opportunities at the intersection of science and software. *The Clinical Neuropsychologist,* 33(2), 271–286. https://doi.org/10.1080/13854046.2018.1535662

Hedge, C., Powell, G., & Sumner, P. (2018). The reliability paradox: Why robust cognitive tasks do not produce reliable individual differences. *Behavior Research Methods*, *50*(3), 1166-1186.

MacLean, P. S., Rothman, A. J., Nicastro, H. L., Czajkowski, S. M., Agurs-Collins, T., Rice, E. L., Courcoulas, A. P., Ryan, D. H., Bessesen, D. H., & Loria, C. M. (2018). The Accumulating Data to Optimally Predict Obesity Treatment (ADOPT) Core Measures Project: Rationale and Approach. Obesity (Silver Spring, Md.), 26 Suppl 2, S6–S15.

Rietveld, C. A., Medland, S. E., Derringer, J., Yang, J., Esko, T., Martin, N. W., … Koellinger, P. D. (2013). GWAS of 126,559 Individuals Identifies Genetic Variants Associated with Educational Attainment. Science, 340(6139), 1467–1471.

Sutin, A. R., Ferrucci, L., Zonderman, A. B., & Terracciano, A. (2011). Personality and obesity across the adult life span. Journal of Personality and Social Psychology, 101(3), 579–592.

Vainik, U., Dagher, A., Realo, A., Colodro-Conde, L., Mortensen, E. L., Jang, K., … Mõttus, R. (2019). Personality-obesity associations are driven by narrow traits: A meta-analysis. Obesity Reviews, 20(8), 1121–1131.

Vainik, U., Neseliler, S., Konstabel, K., Fellows, L. K., & Dagher, A. (2015). Eating traits questionnaires as a continuum of a single concept. Uncontrolled eating. Appetite, 90, 229–239.

Yengo, L., Sidorenko, J., Kemper, K. E., Zheng, Z., Wood, A. R., Weedon, M. N., … Visscher, P. M. (2018). Meta-analysis of genome-wide association studies for height and body mass index in ∼700000 individuals of European ancestry. Human Molecular Genetics, 27(20), 3641–3649.

APPENDIX C: WORKSHOP ORGANIZATIONAL STRUCTURE

CHAIRS
Dana Small, Yale University
Cary Savage, University of Nebraska-Lincoln
Luke Stoeckel, National Institute of Diabetes and Digestive and Kidney Diseases

EXECUTIVE COMMITTEE
Deanna Barch, Washington University in St. Louis
Kerri Boutelle, University of California, San Diego
Lesley Fellows, McGill University
Laura Germine, Harvard University
Barbara Knowlton, University of California, Los Angeles
Russ Poldrack, Stanford University
Cary Savage
Dana Small
Luke Stoeckel

WORKING GROUPS

GENERAL COGNITIVE INDEX
Laura Germine

REWARD AND MOTIVATION
Deanna Barch
John O'Doherty, California Institute of Technology
Brad Appelhans, Rush University
Michael Lowe, Drexel University

ATTENTION AND WORKING MEMORY
Kerri Boutelle
Bob Bilder, University of California, Los Angeles
Vera Maljkovic, Eli Lilly and Company

DECISION MAKING
Lesley Fellows
Daphna Shohamy, Columbia University
Nathaniel Daw, Princeton University
Ifat Levy, Yale University

COGNITIVE CONTROL/EXECUTIVE FUNCTION
Russ Poldrack
Naomi Chaytor, Washington State University
Joel Kramer, University of California, San Francisco
Uku Vainik, University of Tartu (Estonia) and McGill University
Jenny Barnett, Cambridge Cognition
Caitlin Moore, Savonix

LEARNING AND MEMORY

Barbara Knowlton
Neal Cohen, University of Illinois at Urbana-Champaign
Naiman Khan, University of Illinois at Urbana-Champaign
Sara Hargrave, National Institutes of Health Office of Behavioral and Social Sciences Research

SENSATION AND PERCEPTION
Dana Small
Sarah Garfinkel, University of Sussex
Dana Greene-Schloesser, National Institutes of Health Office of Behavioral and Social Sciences Research
Branch Coslett, University of Pennsylvania

AT-LARGE WORKSHOP ATTENDEES
Luke Stoeckel
Cary Savage
Cindy Nowinski, Northwestern University
Jennifer Manly, Columbia University
Mylea Charvat, Savonix
Laura Germine
Marty Sliwinski, Pennsylvania State University
Kimberlee D'Ardenne, Arizona State University
Mark Schatzker

NIH STAFF
Luke Stoeckel
Janine Simmons, National Institute of Mental Health
Todd Horowitz, National Cancer Institute
Dana Greene-Schloesser
Sara Hargrave

REPORT COMMITTEE
Kimberlee D'Ardenne
Dana Small
Cary Savage
Luke Stoeckel
Uku Vainik

APPENDIX D: TASKS NOT RECOMMENDED

1 EXECUTIVE FUNCTION/COGNITIVE CONTROL

The Tower of London task was identified as a high priority measure of higher-order planning and goal-directed behavior. The task has high test-retest reliability, predictive power, is face valid, and available in an open-source format. In the CANTAB Stockings of Cambridge test, the preferred measure, the participant is presented with three balls that must be rearranged to match a particular arrangement according to a set of rules. Rearranging the balls requires planning how to move the balls so that the final arrangement can be achieved. In addition to measuring planning, this task also loads heavily on working memory and general mental effort. However, the researcher is required to purchase CANTAB tasks, which may be a barrier to some users. Therefore, a need is to identify or develop a free, open-source version that approximates the CANTAB Stockings of Cambridge test and retains all the performance features of this task.

https://www.cambridgecognition.com/cantab/cognitive-tests/executive-function/one-touch-stockings-of-cambridge-ots/

2 REWARD/MOTIVATION AND DECISION-MAKING

There is overlap in the roles of reward/motivation and decision-making, and both are felt to be essential areas of emphasis for future obesity and diabetes work, so we have combined for purposes of this section. We make choices in our behaviors that can promote or undermine our health. These choices often require trade-offs and can be influenced by our habits, goals, and how much we value our choice options (Rangel et al., 2008) . There were three tasks identified that may have utility for purposes of this effort. The first two tasks include a two-step sequential learning task (Voon et al., 2015) and a willingness to pay/valuation task like the Becker-DeGroot-Marschak (BDM) auction task (de Berker et al., 2019) or a food valuation task (Tang et al., 2014). While it is felt these measures could have utility in predicting, mediating, and moderating outcomes in obesity and diabetes trials, there is very limited existing research in this area. Finally, we recommend the Episodic Future Thinking task (Stein et al., 2017) as conceptually interesting because it probes an individual's time horizon, which has been shown to modify individual delay discount rates. For this reason, this task has potential direct clinical relevance. Robust psychometric data are lacking, however, and this gap needs to be filled before this task can be recommended for large-scale trials.

3 LEARNING/MEMORY

Tasks that are most likely to be sensitive to factors associated with high BMI involve episodic or relational binding of information. For example, Cheke et al. (2016; 2017) described fMRI and behavioral data in a "What/Where/When" task that assessed the binding of arbitrary relational associations in episodic memory. The effect of BMI on episodic memory was driven by insulin resistance in these young adult participants, which supports the idea that relational, episodic memory measures that assess arbitrary associations will be most sensitive to metabolic dysregulation. Future experimental work should focus on examining the performance of individuals with obesity and/or diabetes on tasks that are specifically sensitive to hippocampal processing. Other experimental work could use more sensitive tests that include retention delays and more trials.

Another area for further exploration is whether factors associated with obesity, such as cardiovascular or endocrine factors, are more specifically associated with memory deficits. For example, Rotenstein et al. (2015) showed that otherwise healthy obese adults showed improvement

on a task of memory for arbitrary associations after treatment with a mineralocorticoid receptor blocker. This finding could implicate mineralcorticoid dysregulation by excessive adipose tissue in hippocampal-dependent memory impairment associated with obesity.

Additionally, behavioral or dietary factors associated with obesity could also lead to memory deficits. Frith el al. (2018) found that performance on the Consortium to Establish a Registry for Alzheimer's Disease (CERAD) word learning test was impaired in older adults who had a poor, pro-inflammatory diet. This task is like some other standardized list learning tasks, where a list of words is given multiple times, assessing learning and memory at a delay. The only group difference on this measure was at the delay, which could be a more episodic measure that requires participants to think back to the study portion of the task. The Frith study is consistent with the idea that some deficits in high BMI participants could be more related to diet/exercise/insulin resistance etc. than obesity per se.

4 SENSATION/PERCEPTION

There are a number of important gaps in the sensation/perception domain. Oral sensation (e.g., taste, flavor, and oral somatosensation) and olfaction are key determinants of food intake and preference. Systematic differences in either sensitivity or affective response to taste, flavors, and food aroma are likely to influence food choice. However, the results from studies associating these perceptions to obesity and diabetes are mixed. Future work is therefore clearly needed.

Because sweet and fat are associated with energy dense foods, sweet and fat perception could influence overeating. However, well-validated and easy-to-administer tests are lacking. To date, large scale studies assessing taste (e.g., NHANES) focus on bitter perception, which have been related to food intake and a number of health outcomes, but associations are mixed. Therefore, work focusing on sweet and fat perception is particularly needed.

Negative associations between visceral fat and olfactory sensitivity have also been reported, but additional research is needed. A short olfactory test focusing on response to food odors would be a useful instrument for further elucidating links between smell perception and obesity and diabetes.

There is mixed evidence regarding associations between taste and smell perception and obesity and diabetes. In addition, commercially available assessments are expensive and specialized equipment is required to create taste and smell stimuli. Taste and smell assessment was therefore not recommended for inclusion in the batteries.

There are well-studied and reliable measures of the perception of and satisfaction with one's body, such as the Body Shape Questionnaire (Cooper et al., 1987). However, the extent to which body perception is related to diabetes, eating behavior, and obesity is unclear but is a potentially interesting avenue for future research.

Body schema, referring to the integrated on-line mental representation of the body, was also considered because of the evidence of altered body image in eating disorders and obesity (Cash and Deagle, 1997; Gardner et al., 1987). However, the paucity of evidence regarding obesity and body schema perception argued against its inclusion.

Because chronic pain is highly co-morbid with obesity (Stone and Broderick, 2012), and experienced pain is associated with weight gain and difficulty with weight loss (Masheb et al., 2015), an assessment of pain is recommended for inclusion in future batteries. Currently, the short-form McGill Pain Questionnaire is the most commonly used tool to assess pain. It is easily administered and has

high reliability in a variety of populations. However, because this measure is self-report rather than a direct assessment of pain, the majority opinion of the working group was to leave pain assessment out of the initial neuropsychological batteries.

Visual spatial ability, primarily assessed with the Rey-Osterrieth complex figure, has also been consistently found to be impaired in obesity (Boeka and Lokken, 2008; Eneva et al., 2017). The test includes a copy phase followed by an immediate and delayed recall. Importantly, deficits emerge on the copy, pointing to visual spatial, rather than memory impairment (Boeka and Lokken, 2008). However, this test also taps executive function (e.g., Shin et al., 2006), which is clearly impaired in obesity. The Penn Line Orientation Task (PLOT) is a pure measure of visual spatial ability (Moore et al., 2015) and emerging findings from the human connectome project data suggest that it is strongly associated with BMI independent of socioeconomic status, education, age, and gender in a large cohort of healthy individuals (Vainik et al., 2018). Because of the associations identified in the human connectome project data, we recommend this task for inclusion in the Extended Battery. However, there are currently no studies linking visual spatial abilities with outcomes on weight-loss trials. The abbreviated version of the PLOT requires 5 min. The PLOT item responses are coded in two ways: (1) dichotomous, such that the rotation to a perfect parallel line is 1 and all deviations are 0; and (2) polytomous, such that rotation to a perfectly parallel line set receives the maximum score of 3, and each mouse click away from perfectly parallel decreases the score by 1.

Additional references in Appendix D.

de Berker, A. O., Kurth-Nelson, Z., Rutledge, R. B., Bestmann, S., & Dolan, R. J. (2019). Computing Value from Quality and Quantity in Human Decision-Making. *Journal of Neuroscience*, *39*(1), 163-176.

Boeka, A. G., & Lokken, K. L. (2008). Neuropsychological performance of a clinical sample of extremely obese individuals. *Archives of Clinical Neuropsychology*, *23*(4), 467-474.

Cash, T. F., & Deagle III, E. A. (1997). The nature and extent of body-image disturbances in anorexia nervosa and bulimia nervosa: A meta-analysis. *International Journal of Eating Disorders*, *22*(2), 107-126.

Cheke, L. G. (2016). What–where–when memory and encoding strategies in healthy aging. *Learning & Memory*, *23*(3), 121-126.

Cheke, L. G., Bonnici, H. M., Clayton, N. S., & Simons, J. S. (2017). Obesity and insulin resistance are associated with reduced activity in core memory regions of the brain. *Neuropsychologia*, *96*, 137-149.

Cooper, P. J., Taylor, M. J., Cooper, Z., & Fairbum, C. G. (1987). The development and validation of the Body Shape Questionnaire. *International Journal of eating disorders*, *6*(4), 485-494.

Eneva, K. T., Murray, S. M., & Chen, E. Y. (2017). Binge-eating disorder may be distinguished by visuospatial memory deficits. *Eating behaviors*, *26*, 159-162.

Frith, E., Shivappa, N., Mann, J. R., Hébert, J. R., Wirth, M. D., & Loprinzi, P. D. (2018). Dietary inflammatory index and memory function: population-based national sample of elderly Americans. *British Journal of Nutrition*, *119*(5), 552-558.

Supplementary Material

Gardner, R. M., Martinez, R., & Sandoval, Y. (1987). Obesity and body image: an evaluation of sensory and non-sensory components. *Psychological Medicine*, *17*(4), 927-932.

Masheb, R. M., Lutes, L. D., Kim, H. M., Holleman, R. G., Goodrich, D. E., Janney, C. A., ... & Damschroder, L. J. (2015). Weight loss outcomes in patients with pain. *Obesity*, *23*(9), 1778-1784.

Moore, T. M., Scott, J. C., Reise, S. P., Port, A. M., Jackson, C. T., Ruparel, K., ... & Gur, R. C. (2015). Development of an abbreviated form of the Penn Line Orientation Test using large samples and computerized adaptive test simulation. *Psychological assessment*, *27*(3), 955.

Rangel, A., Camerer, C., & Montague, P. R. (2008). A framework for studying the neurobiology of value-based decision making. *Nature reviews neuroscience*, *9*(7), 545.

Rotenstein, L. S., Sheridan, M., Garg, R., & Adler, G. K. (2015). Effect of mineralocorticoid receptor blockade on hippocampal-dependent memory in adults with obesity. *Obesity*, *23*(6), 1136-1142.

Shin, M. S., Park, S. Y., Park, S. R., Seol, S. H., & Kwon, J. S. (2006). Clinical and empirical applications of the Rey–Osterrieth complex figure test. *Nature protocols*, *1*(2), 892.

Stone, A. A., & Broderick, J. E. (2012). Obesity and pain are associated in the United States. *Obesity*, *20*(7), 1491-1495.

Tang, D. W., Fellows, L. K., & Dagher, A. (2014). Behavioral and neural valuation of foods is driven by implicit knowledge of caloric content. *Psychological science*, *25*(12), 2168-2176.

Voon, V., Derbyshire, K., Rück, C., Irvine, M. A., Worbe, Y., Enander, J., ... & Robbins, T. W. (2015). Disorders of compulsivity: a common bias towards learning habits. *Molecular psychiatry*, *20*(3), 345.

APPENDIX E: TEST-RETEST RELIABILITIES AND POWER CALCULATION TOOL

Find the link to the Excel spreadsheet in the Supplemental materials on the *NutriXiv* landing page. https://osf.io/j26vy/
